# Osteoprotegerin: Relationship to Breast Cancer Risk and Prognosis

**DOI:** 10.3389/fonc.2020.00462

**Published:** 2020-04-07

**Authors:** Dirk Geerts, Christina Chopra, Linda Connelly

**Affiliations:** ^1^Department of Medical Biology, Academic Medical Center Amsterdam, Amsterdam, Netherlands; ^2^School of Medicine, California University of Science and Medicine, San Bernardino, CA, United States

**Keywords:** osteoprotegerin (OPG), TNFRSF11B, breast cancer, risk, prognosis, TNF superfamily

## Abstract

Osteoprotegerin (OPG) is a secreted member of the Tumor Necrosis Factor (TNF) receptor superfamily (TNFRSF11B), that was first characterized and named for its protective role in bone remodeling. In this context, OPG binds to another TNF superfamily member Receptor Activator of NF-kappaB Ligand (RANKL; TNFSF11) and blocks interaction with RANK (TNFRSF11A), preventing RANKL/RANK stimulation of osteoclast maturation, and bone breakdown. Further studies revealed that OPG protein is also expressed by tumor cells and led to investigation of the role of OPG in tumor biology. An increasing body of data has demonstrated that OPG modulates breast tumor behavior. Initially, research was focused on OPG in the bone microenvironment as a potential inhibitor of RANKL-driven osteolysis. More recently, attention has shifted to include OPG expression and interactions in the primary breast tumor independent of RANKL. In the primary tumor, OPG may interact with another TNF superfamily member, TNF-Related Apoptosis Inducing Ligand (TRAIL; TNFSF10) to prevent apoptosis induction. Additional interest in OPG in breast cancer has been stimulated by the tumor-promoting role of its binding partner RANKL in association with BRCA1 gene mutations. We and others have previously summarized the functional studies on OPG and breast cancer (1, 2). After basic research studies on the *in vitro* role for OPG (and RANKL) in breast cancer, the field now expands to assess the *in vivo* role for OPG by examining the correlation between OPG expression and breast cancer risk or patient prognosis. However, the data reported so far is conflicting, since OPG expression appears linked to both good and poor patient survival. In the current review we will summarize these studies. Our goal is to provide stimulus for further research to bridge the basic research findings and clinical data regarding OPG in breast cancer.

## Introduction

Osteoprotegerin (OPG; encoded by the TNFRSF11B gene) is a secreted member of the TNF receptor protein superfamily that was first characterized and named for its protective role in bone remodeling (3, 4). OPG acts as a decoy receptor for Receptor Activator of NF-kappaB Ligand (RANKL; TNFSF11), blocking interaction with RANK (TNFRSF11A) and thereby stimulation of osteoclast maturation. It was subsequently discovered that OPG is expressed by breast tumor cells, including human cancer cell lines and tissue samples (5). OPG also interacts with another TNF superfamily member, TNF-Related Apoptosis Inducing Ligand (TRAIL; TNFSF10), blocking induction of apoptosis (5, 6). In the 15 years since the first report of OPG expression in breast tumor tissue, a growing number of studies have investigated a mechanistic role for OPG in breast tumorigenesis. These studies have been previously reviewed by our group as well as others (1, 2, 7). Here we will focus on the clinical studies regarding OPG expression and links to breast cancer risk and prognosis in breast cancer patients. Clarifying the role of OPG in breast cancer patients will validate current research findings and define the questions for future mechanistic studies.

## OPG Levels and Breast Cancer Risk

Three types of studies with regards to OPG and breast cancer risk exist. One type has focused on Single Nucleotide Polymorphisms (SNPs) in the OPG gene in association with breast cancer. Another type of study has determined whether OPG protein levels correlate with breast cancer risk. The third type of study has investigated OPG protein levels and breast cancer risk in association with BRCA mutations. The findings from each of these studies are described below.

### SNPs and Breast Cancer Risk

TNFRSF11B/OPG is a single copy gene located on chromosome 8q23-34, that spans 29 kb and consists of 5 exons (8). Studies on the OPG gene and breast cancer risk have focused on 3 SNPs – rs3102735, rs2073617, and rs2073618 (for all SNP annotations see https://www.ncbi.nlm.nih.gov/snp/). The rs3102735 SNP (major allele T, minor allele C) and rs2073617 SNP (major allele T, minor allele C) are in the 5′ promoter region of the OPG gene (9, 10). The rs2073618 OPG SNP (major allele G, minor allele C) is in the first exon of OPG with the minor allele C causing the third amino acid in the OPG protein to change from Lysine to Asparagine (11).

Ney et al. (12) studied the frequency of two OPG gene SNPs (rs3102735 and rs2073618) in 614 breast cancer patients and 784 healthy subjects. They found that the minor allele C of SNP rs3102735 was associated with a 1.5-fold increased risk for breast cancer. They did not find an association between the OPG gene SNP rs2073618 and breast cancer risk, but did establish that the major allele G was more likely to be found in invasive vs. non-invasive breast cancer cases (12). Ney et al. did not measure OPG protein serum levels, but studies on the same SNPs for different disease states did not find differences in OPG serum levels associated with SNPs rs3102735 or rs2073618 (13, 14).

A second group investigated associations of OPG gene SNPs rs2073618 and rs2073617 with breast cancer in 176 breast cancer patients and 100 healthy subjects. Unlike the study by Ney et al. described above, this study found that the SNP rs2073618 minor allele C was more frequent in breast cancer patients than in the control group (15). OPG serum levels in breast cancer patients and healthy subjects were not associated with SNP rs2073618 (15). An increased frequency of the major T allele in the OPG gene SNP rs2073617 was also observed in breast cancer patients as compared to the control group, again with no significant difference in OPG serum levels. These findings on SNPs rs2073618 and rs2073617 allele frequency and increased breast cancer risk were confirmed in a recent study of 115 breast cancer patients and 120 healthy subjects (16). In addition, a combined genotype of heterozygous for the GG major allele for OPG rs2073618 and the CC minor allele for OPG rs2073617 was shown to be protective against the breast cancer (16).

In summary, these studies demonstrate that OPG gene SNPs are associated with breast cancer risk (Table 1). The lack of differences in OPG serum levels would suggest that the effect of SNPs may be related to a change in protein activity, not expression level. However, additional studies on genotype and subsequent phenotype are required before conclusions can be drawn as to pro- or anti-tumor effects of OPG SNPs.

**Table 1:** Summary of studies regarding SNPs in the OPG gene and links to breast cancer.

**Study**	**Genomic DNA from**	**SNP analysis and findings**
Ney et al. (12)	614 breast cancer patients 784 healthy subjects	*rs3102735*- Minor C allele, 1.5x increased risk for breast cancer *rs2073618*- No association
Omar et al. (15) Shaker et al. (16)	176 breast cancer patients 100 healthy subjects 115 breast cancer patients 120 healthy subjects	*rs2073618*- Minor C allele more frequent in breast cancer patients *rs2073617*- Major T allele more frequent in breast cancer patients

### OPG Protein Serum Levels and Breast Cancer Risk

OPG serum levels and breast cancer risk were initially examined in a large study which recruited 6,279 subjects (male and female) in Norway with no previous history of cancer (17). Serum samples were collected from each participant and frozen for the duration of the study (12 years) before measurement of OPG by ELISA. Nine hundred forty-eight participants developed cancer, of which 76 got breast cancer. Thirty cases of breast cancer were reported in the 1st Tertile of OPG serum group (0.46–2.78 ng/ml), 26 breast cancer cases for the 2nd Tertile of OPG serum group (2.79–3.55 ng/ml) and 20 breast cancer cases for the 3rd Tertile of OPG serum group (3.56–25.81 ng/ml). Based on this, it was calculated that women in the upper Tertile of serum OPG had a 45% lower relative risk of breast cancer compared to women in the 1st Tertile. Further analysis revealed that there was a 76% reduction in relative risk with high OPG levels when samples were analyzed in women below 60 years of age.

OPG expression vs. breast cancer risk has been analyzed in a more recent, similar type of study (18). The European Prospective Investigation into Cancer and Nutrition (EPIC) cohort was a study designed to identify risk factors for cancer. From the 235,607 women who participated in this study, 2008 serum samples were analyzed for OPG expression from women who subsequently developed breast cancer alongside a group of matched healthy subjects. Of the 2008 cancer cases, 81% were Estrogen Receptor positive (ER+) and 19% were ER negative (ER–). The samples were organized into Tertiles based on the OPG serum levels determined at the start of the study before patients developed breast cancer: T1 = < 0.18 ng/ml, T2 = 0.18– < 0.22 ng/ml, and T3 = ≥ 0.22 ng/ml. Women with higher OPG serum levels had an increased relative risk (1.93) of risk for ER- breast cancer (T1–82, T2–78, T3–98 cases). There was a modest reduction in risk (0.84) of ER+ breast cancer with high OPG (T1–342, T2–297, T3–290 cases). When comparing these data with Vik et al. it appears that the range of OPG values are much lower in the Fortner et al. study (17, 18). The Vik et al. study. Tertiles look at OPG levels from 0.46 to 25.81 ng/ml while the Fortner et al. study looks at OPG levels from 0.18 ng/ml and below to above 0.22 ng/ml. The Fortner study reports a difference between ER+ and ER– samples, while ER status was not assigned in the Vik et al. study. Based on common findings, it is likely that the predominant subtype in 76 patients in the Vik et al. study would be ER+, and then there would be agreement in the association of low OPG serum levels with reduced risk for ER+ breast cancer (19).

In the only other study examining breast cancer risk, OPG serum levels were measured in a group of 278 postmenopausal women (20). The group was subsequently stratified as to whether they developed breast cancer within 12 months (40 women) or within 12–24 months (58 women), while the women that did not develop breast cancer during follow up were used as healthy subjects (180 women). Serum OPG levels ranged from 0.60–9.91 pM. There were no differences in serum OPG levels in the women who developed breast cancer within 12–24 months as compared to healthy subjects. However, in the group that developed breast cancer within 12 months, serum OPG levels were higher than in healthy subjects. It should be noted that tumor development in the 12 months prior to detection could lead to OPG protein production by tumor cells. Therefore, this data may not necessarily reflect OPG levels and “breast cancer risk”.

Another study followed up on women in the EPIC cohort who developed breast cancer and analyzed risk of death following a breast cancer diagnosis in relation to pre-diagnosis OPG serum levels and ER subtype in 2006 women (21). There was an increased risk of breast cancer-specific mortality in women with ER+ disease who had higher pre-diagnosis OPG serum levels (Quintile 5 > 12.38 pM as compared to Quintile 1 ≤ 7.80 pM). OPG levels were not associated with mortality risk in women with ER- breast cancer. Therefore, while the previous study by this group in the EPIC cohort linked high OPG with a slightly reduced risk for ER+ breast cancer, after breast cancer development, higher OPG levels correlate with poorer prognosis in this patient group.

### OPG and Breast Cancer Risk With BRCA Mutations

RANKL plays a role in the breast cancer development signaling in patients with BRCA1 mutations (22). The ability of OPG to bind to RANKL and block its activity raised interest in the role of OPG in BRCA1-mutated breast cancer. Serum OPG levels were measured in 391 BRCA1 or−2 mutation carriers and 782 non-carrier healthy subjects. BRCA mutation carriers were found to have lower OPG serum levels than healthy subjects [range of values studied were 10.8 to 1,414 pg/ml (23)]. This study did not include a follow up to determine whether low OPG levels linked to breast cancer risk.

In a subsequent study, 206 women with BRCA1 or−2 mutations had serum OPG levels measured, were divided into low OPG (mean 62.9 pg/ml; range 4.2–94.5 pg/ml) or high OPG (mean 168.1 pg/ml; range 95.5–547.7 pg/ml) groups, and were followed for the development of breast cancer. Within 6.5 years, 13 of 103 women in the low OPG group developed breast cancer, compared with 6 of 103 women in the high OPG group (23). Overall, the women who developed breast cancer had lower baseline OPG serum levels (mean 90.59 pg/ml; range 4.2–205.7 pg/ml) compared to women who did not develop breast cancer (mean 117.9 pg/ml; range 7.4–547.7 pg/ml). The authors acknowledged that this was a small study, and that the effects observed were only marginally significant.

In summary, the limited studies in this area so far do not establish a clear link between OPG serum levels and breast cancer risk in BRCA mutation carriers. Additional studies are needed to establish whether these are indeed linked. The implications of these studies also need to be considered in light of the high breast cancer risk in this patient population.

In the studies on breast cancer risk we discussed, OPG expression was characterized as serum OPG levels. These studies are summarized in Table 2. In marked contrast, the studies that analyzed OPG expression in association with prognosis described below mainly consider OPG expression in the primary breast tumor.

**Table 2:** Summary of studies investigation associations between OPG and breast cancer risk.

**Study**	**Participants**	**OPG Analysis**	**Significant data**
Vik et al. (17)	76 women	Serum	Reduced breast cancer risk with high OPG expression
Kiechl et al. (20)	278 post-menopausal women	Serum	High OPG expression associated with breast cancer development within 12 months
Fortner et al. (18)	2008 women (EPIC cohort)	Serum	High OPG expression associated with increased risk for ER- breast cancer, suggestive inverse association for ER+
Sarink et al. (21)	2006 women (EPIC cohort)	Serum	High OPG expression associated with increased mortality in ER+ breast cancer
Widschwendter et al. (24)	391 BRCA1/2 mutation carriers 782 healthy subjects	Serum	BRCA mutation carriers had lower OPG expression
Oden et al. (23)	206 BRCA1/2 mutation carriers	Serum	Lower OPG expression in women that later developed breast cancer

## OPG Expression and Breast Cancer Patient Prognosis

The initial studies on OPG expression in patient samples were performed by IHC (immunohistochemistry) on breast tumor tissues. In a study of 400 patient samples, lower OPG protein expression was observed with increasing tumor grade (5, 25). OPG protein expression was higher in ER+ samples than in ER–samples (5, 25, 26).

Subsequent studies on OPG expression in breast cancer patients were performed using microarray data of OPG mRNA expression in breast cancer tissues. Santini et al. examined OPG expression in a publicly available dataset of 295 primary breast cancer patients (27). In contrast to the earlier work by Holen et al. this study found no significant difference in OPG mRNA expression between ER+ and ER– tissue samples. There was also no difference in OPG mRNA expression in relation to primary tumor size (< or > 2 cm). However, in agreement with the earlier immunohistochemical data, lower OPG mRNA expression was observed with increasing tumor grade. This study also separated patients into “good” and “poor” prognosis groups and found that there was a significant association of higher OPG mRNA expression with “good” prognosis. Furthermore, Kaplan-Meier analysis showed that patients with high primary tumor OPG mRNA expression had increased disease free survival (DFS) and overall survival (OS).

Owen et al. analyzed OPG mRNA expression by quantitative RT-PCR in 127 breast cancer tissues and 31 non-neoplastic control breast tissues (28). They did not find significant differences in OPG mRNA expression in tumor vs. normal samples or in ER+ vs. ER– samples. Kaplan-Meier analyses, in contrast to the Santini et al. data, showed that patients with higher OPG mRNA expression had significantly poorer OS (108 vs. 142 months).

These initial studies show varying effects. Further studies on OPG expression and breast cancer prognosis share this pattern. Indeed, having found discordant results in microarray analysis for OPG mRNA expression in a small number of breast cancer data sets, Sanger et al. expanded their study to 40 publicly available breast cancer microarray datasets [4,467 samples; (29)]. The patient samples with high OPG mRNA expression levels were less likely to be ER+, but more likely to be Progesterone Receptor positive. This is in contrast to the study by Holen et al. that linked high OPG expression with ER+ samples by IHC (5). OPG expression was associated with a better prognosis in ER+ tumors but there was no significant prognostic value for OPG in the ER- group (29). It should be noted that when the datasets were studied individually, only 7 of 18 ER+ groups showed a significant effect or trend for better prognosis with high OPG, 11 of 18 showed no difference in patient survival.

Park et al. studied 175 breast tumor tissue samples by IHC on tissue microarray blocks and stratified into patients with (85 patients) and without (90 patients) tumor OPG expression (30). In agreement with the previous two studies that linked a reduced OPG expression levels with increasing tumor grade, patients with larger tumors (> 2 cm) or T4 tumors [tumors of any size growing into the chest wall or skin; (31)] were less likely to have tumor OPG expression than patients with smaller tumors. A lack of OPG expression was also associated with lymph node involvement and higher proliferation index as measured by Ki67 staining. Patients with OPG-expressing tumors were more likely to undergo breast-conserving surgery (as opposed to mastectomy) than those with OPG-negative tumors. Despite these suggestions that OPG expression was correlated to good prognosis, no link was found between OPG expression and disease free or OS or ER status.

Two studies by Labovsky et al. (32, 33) examined OPG expression by IHC in 63 tissue biopsies from patients with breast cancer. They found that OPG expression was higher in the breast tumors as compared to 10 non-neoplastic control breast tissues (32). In addition, OPG expression in spindle-shaped stromal cells within the tumors was associated with metastasis (33). The authors proposed that the spindle-shaped cells were tumor-recruited mesenchymal stem cells with metastasis-promoting function.

Luo et al. performed a study using publicly available breast tumor microarray data (546 samples) and breast tumor biopsies [86 samples; (34)]. Kaplan Meier analysis showed that high OPG expression was associated with increased OS and increased distant metastasis-free survival in both data sets. No significant effect was observed for OPG expression in relation to lung metastasis-free survival. IHC on the breast tumor biopsies showed that OPG expression was down-regulated in breast tumors as compared with adjacent normal tissue.

In another study on publicly available tumor sample data, Vidula et al. used microarray analysis on pre-treatment biopsies taken from 149 patients in the I-SPY1 trial (patients with at least 3 cm invasive breast cancer with no distant metastases) and a dataset from 425 pre-treatment biopsies (35). In the I-SPY1 tissue samples, OPG expression was higher in HER2– vs. HER2+ samples. OPG expression was also higher in Stage I/II than in Stage III and inflammatory tumors, and higher in Grade I than in Grade II/III tumors. However, they found no significant correlation between OPG expression and RFS (relapse free survival) in either data set.

Timotheadou et al. performed quantitative RT-PCR on 814 breast cancer tissue samples before adjuvant therapy (36). They found high OPG mRNA expression was associated with the Luminal A breast cancer subtype. Low OPG mRNA expression was associated with higher histological tumor grade, and with radical mastectomy. This study did not identify a significant association between OPG expression and DFS or OS.

While the studies discussed so far have analyzed OPG mRNA or protein expression in the breast tumors in association with prognosis, a recent study has looked at OPG serum levels in breast cancer patients. Rachner et al. measured serum OPG in 504 patients with primary, non-metastatic breast cancer (37). They found that high serum OPG levels were associated with lower survival in Kaplan Meier analysis. The mean serum OPG levels in this study were 4.24 ± 1.68 pM (range 0.46–13.40 pM) and 81% of the patients had ER+ disease. Given the ability of OPG to exert a “protective” effect in bone, the authors also examined the presence of bone metastases. They found no association between serum OPG levels and bone metastasis development.

## Discussion

The studies published so far present varied conclusions: 3 studies find OPG expression associated with better prognosis, 3 find OPG expression associated with poor prognosis, and 3 found no link between OPG and prognosis (Table 3). However, it should be noted that 5 of the studies reported that OPG tumor expression decreased with increasing tumor grade as the most consistent finding across the datasets. Future studies may want to take this characteristic into account when analyzing their data. There is currently limited understanding regarding regulation of OPG expression in breast cancer, and the mechanism by which OPG expression changes with tumor grades is unknown. We have reported that there is an increase in OPG gene copy number in breast cancers and that this increase correlates with poor survival (38). In addition, we have shown that OPG expression is up-regulated in breast cancer cells co-cultured with macrophages and this is in part due to the presence of Interleukin 1Beta (39). These studies demonstrate the potential for increased OPG expression in breast cancer, but the mechanisms for down-regulation with increasing tumor grade require further investigation.

**Table 3:** Summary of studies regarding OPG and breast cancer prognosis.

**Study**	**Tissues/Subjects**	**OPG analysis**	**Significant data**
Holen et al. (5) and Cross et al. (25)	400 breast tumors	Protein IHC	•OPG expression lower with increasing tumor grade •OPG expression lower in ER- than ER+ tumors
Van Poznak et al. (26)	40 breast tumors	Protein IHC	•Correlation between OPG expression and ER/PR status
Santini et al. (27)	295 breast tumors	RNA	•OPG expression associated with good prognosis
OPG = good prognosis		Microarray	•High OPG expression associated with increased DFS and OS •No difference ER+ vs. ER-
Owen et al. (28)	127 breast tumors	RNA	•High OPG expression associated with significantly poorer OS
OPG = poor prognosis	31 matching normal tissues	qRT-PCR	
Sanger et al. (29)	4,467 breast tumors	RNA	•OPG expression lower in ER+
OPG = good prognosis (ER+)		Microarray	•OPG expression associated with better prognosis in ER+
Park et al. (30)	175 breast tumors	IHC	•Lower OPG expression with increasing tumor grade
No link			•OPG expression associated with lack of lymph node involvement and lower proliferation index •No link OPG expression with DFS/OS
Labovsky et al. (32, 33)	63 breast tumors	IHC	•Higher OPG expression in breast tumors than in healthy tissue
OPG associated with metastasis	10 non-neoplastic control tissues		•OPG expression in spindle-shaped stromal cells from breast tumors associated with presence of metastasis
Luo et al. (34)	546 breast tumors dataset	RNA	•High OPG expression associated with increased OS and distant metastasis free survival
OPG = good prognosis	86 breast tumors	Microarray IHC	•IHC showed OPG down-regulated in breast tumors compared to adjacent tissue
Vidula et al. (35)	149 I-SPY1 breast tumors	RNA	•Higher OPG expression in HER2– vs. HER+
No link	425 breast tumor dataset (all pre-treatment)	Microarray	•Higher OPG expression in lower stage and grade of tumor •No significant effect on relapse free survival
Timotheadou et al. (36)	814 breast tumors	RNA	•Higher OPG expression associated with luminal A subtype
No link		qRT-PCR	•Low OPG expression associated with modified radical mastectomy and higher histological grade •No association between OPG and DFS/OS
Rachner et al. (37)	504 primary, non-metastatic breast cancer patients	Serum	•High OPG serum levels associated with poorer survival
OPG = poor prognosis			

*Studies which present data in relation to prognosis are highlighted as good prognosis (yellow), poor prognosis (blue), and no link to prognosis (green)*.

The majority of the studies on OPG expression and prognosis have analyzed mRNA expression. Recently Rachner et al. took a different approach and measured OPG serum levels (37). Here they found that high OPG serum levels correlated with reduced survival for breast cancer patients. This data agrees with the breast cancer risk studies which found that high serum OPG levels before breast cancer diagnosis was associated with increased risk as well as higher mortality following diagnosis. Further measurements of OPG serum levels are required to validate prognostic effects once breast cancer has developed.

Several of the studies observe differences in effects of OPG in relation to ER+ or ER– subtypes. Only limited mechanistic studies exist on the link between OPG expression and ER status. Treatment of ER+ breast cancer cells with 17Beta-Estradiol *in vitro* resulted in decreased OPG mRNA and protein expression (40). We analyzed OPG mRNA expression in datasets from breast cancer cell lines and found that OPG expression was higher in basal as compared to luminal cell lines (38). Further analysis is required to determine the association between OPG expression and ER status in breast tumor samples.

An analysis of the variable results of studies into the role of OPG in breast cancer risk and prognosis must also reflect on the current status of the signal transduction literature. OPG has the potential to interact with RANKL, which exerts primary tumor-promoting and metastasis-promoting roles in breast cancer (3, 4, 41, 42). OPG-mediated blocking of RANKL signaling in breast cancer would likely have an anti-tumor effect and result in a better prognosis. However, OPG has also been described to interact with TRAIL and thereby prevent induction of apoptosis in tumor cells (5, 6). In this context, OPG would have a tumor-promoting effect and thus be associated with a poorer prognosis. OPG may also interact with other, as yet uncharacterized, ligands, and receptors within the breast tumor microenvironment. OPG must therefore also be considered in an environmental context – actions of OPG in the primary tumor have been reported to be tumor-promoting while studies that focus on the bone microenvironment show anti-tumor effects (38, 39, 42–44). In a previous study, our group has inoculated OPG knockdown cells in a chick embryo metastasis model and observed a reduction in the ability of cells to metastasize to the chick tissues (38). In contrast, administration of recombinant OPG-Fc reduced the growth of bone tumors in a murine model (44). Interestingly, these contrasting effects of OPG were illustrated in a study by Zinonos et al. where OPG-overexpressing breast cancer cells were introduced in a murine model by intra-tibial injection. While the overexpression of OPG limited tumor growth and bone loss in the bone microenvironment, there was a significant increase in the incidence of pulmonary metastasis (45). Given these opposing effects, it is likely that OPG expression could both be a benefit and a risk to the breast cancer patient, possibly with the opposing effects occurring at different stages and different sites in the disease process. Thus, the varying correlations of OPG with prognosis, as reported this far, are perhaps not so surprising. Therefore, we conclude that further investigation is needed on the biological effects of OPG at different stages of breast cancer and that an integration of the signaling and clinical study data is necessary to fully understand the role of OPG in breast cancer.

## Author Contributions

DG and LC conceptualized the manuscript, conducted literature searches, analyzed and interpreted the literature and drafted the manuscript. CC conducted literature searches, categorized the papers and assisted with editing the manuscript. All authors read and approved the manuscript.

### Conflict of Interest

The authors declare that the research was conducted in the absence of any commercial or financial relationships that could be construed as a potential conflict of interest.
